# Long-Term Cochlear Implant Outcomes in Children with *GJB2* and *SLC26A4* Mutations

**DOI:** 10.1371/journal.pone.0138575

**Published:** 2015-09-23

**Authors:** Che-Ming Wu, Hui-Chen Ko, Yung-Ting Tsou, Yin-Hung Lin, Ju-Li Lin, Chin-Kuo Chen, Pei-Lung Chen, Chen-Chi Wu

**Affiliations:** 1 Department of Otolaryngology—Head and Neck Surgery, Chang-Gung Memorial Hospital, Linkou Branch, College of Medicine, Chang-Gung University, Taoyuan, Taiwan; 2 Department of Otolaryngology, National Taiwan University Hospital, Taipei, Taiwan; 3 Graduate Institute of Medical Genomics and Proteomics, National Taiwan University College of Medicine, Taipei, Taiwan; 4 Division of Genetics and Endocrinology, Department of Pediatrics, Chang-Gung Memorial Hospital, Linkou Branch, College of Medicine, Chang-Gung University, Taoyuan, Taiwan; 5 Department of Medical Genetics, National Taiwan University Hospital, Taipei, Taiwan; Innsbruck Medical University, AUSTRIA

## Abstract

**Objectives:**

To investigate speech and language outcomes in children with cochlear implants (CIs) who had mutations in common deafness genes and to compare their performances with those without mutations.

**Study Design:**

Prospective study.

**Methods:**

Patients who received CIs before 18 years of age and had used CIs for more than 3 years were enrolled in this study. All patients underwent mutation screening of three common deafness genes: *GJB2*, *SLC26A4* and the mitochondrial 12S rRNA gene. The outcomes with CIs were assessed at post-implant years 3 and 5 using the Categories of Auditory Performance (CAP) scale, Speech Intelligibility Rating (SIR) scale, speech perception tests and language skill tests.

**Results:**

Forty-eight patients were found to have confirmative mutations in *GJB2* or *SLC26A4*, and 123 without detected mutations were ascertained for comparison. Among children who received CIs before 3.5 years of age, patients with *GJB2* or *SLC26A4* mutations showed significantly higher CAP/SIR scores than those without mutations at post-implant year 3 (p = 0.001 for CAP; p = 0.004 for SIR) and year 5 (p = 0.035 for CAP; p = 0.038 for SIR). By contrast, among children who received CIs after age 3.5, no significant differences were noted in post-implant outcomes between patients with and without mutations (all p > 0.05).

**Conclusion:**

*GJB2* and *SLC26A4* mutations are associated with good post-implant outcomes. However, their effects on CI outcomes may be modulated by the age at implantation: the association between mutations and CI outcomes is observed in young recipients who received CIs before age 3.5 years but not in older recipients.

## Introduction

Congenital sensorineural hearing loss (SNHL) has an incidence of approximately 0.1% in live births [1]. At least 50% of these cases are hereditary, and approximately 70–80% of cases of genetic deafness are non-syndromic, where deafness is not associated with any other clinical features [2,3]. Mutations in three genes, including *GJB2* (or *CX26*, Gene ID: 2706) [4], *SLC26A4* (or *PDS*, Gene ID: 5172) [5] and the mitochondrial 12S rRNA gene (*MTRNR1*) [6], have been reported to be highly prevalent in SNHL patients across different populations. It has been estimated that approximately 30–50% of idiopathic SNHL cases are attributable to mutations in these three genes [7,8].

With the restoration of hearing via a cochlear implant (CI), auditory and oral performances in children with severe-to-profound SNHL have been significantly improved [9]. However, their post-implant outcomes are highly variable. In addition to factors such as age at implantation and duration of implant use [9,10], different genetic etiologies might have an impact on CI outcomes as well (see Table 1). Implanted children with *GJB2* mutations were reported to score higher than those without *GJB2* mutations on measures of auditory performance and speech production [11,12], word and sentence perception [13], reading comprehension [14,15], and expressive language [16]. A study showed that children with *SLC26A4* mutations demonstrated better results of speech perception and production than those with an unknown etiology, although statistical significance was not reached [11]. In our previous studies, children with mutations in any of the three common deafness genes displayed better auditory performance after 3 years of CI use [17,18].

**Table 1:** Review of studies on language/speech outcomes in cochlear implanted patients with mutations in *GJB2*, *SLC26A4* or the mitochondrial 12S rRNA gene.

Study	No. of subjects with *GJB2*, *SLC26A4*, Mito. 12S rRNA mutations	Age at CI (mean)	Length of CI use at test (mean) or test time point(s)	Measures	Results
Yan et al.[11]	15, 10, n/a	0.8–5 (2.3) y	1, 2 y	MAIS; CAP; SIR	Better in patients with *GJB2* mutations, but not *SLC26A4* mutations, at year 2
Matsushiro et al.[12]	4, n/a, n/a	3.0–5.8 (3.8) y	0.4–7.1 (1.6) y	IT-MAIS	Better in patients with *GJB2* mutations
Sinnathuray et al.[13]	11, n/a, n/a	2.5–10.3 y	3 y	IOWA Matrix; GASP	No difference in IOWA Matrix; better GASP in patients with *GJB2* mutations
Green et al.[14]	8, n/a, n/a	n/a	> 3 y	Speech perception	Better in patients with *GJB2* mutations
Bauer et al.[15]	22, n/a, n/a	< 5 y	0–0.5 y	A battery of measures	Better nonverbal cognition and reading comprehension in patients with *GJB2* mutations
Wu et al.[17]	4, 18, 0	1–14 (4.7) y	3–10 (4.4) y	Speech perception	Better in patients with *SLC26A4* mutations
Wu et al.[18]	12, 22, 1	5.0 ± 2.8 y	3 y	CAP	Better in patients with *GJB2* or *SLC26A4* mutations
Cullen et al.[19]	20, n/a, n/a	3.3 ± 2.9 y	1, 2, 3 y	Speech perception	No difference
Davcheva-Chakar et al.[20]	7, n/a, n/a	2.5–5.6 (4.4) y	1, 2 y	Speech perception	No difference
Yoshida et al.[21]	9, 2, n/a	1.8–5.3 (3.1) y	3.9–5.2 (4.7) y	IT-MAIS; speech perception under noise	No difference
Karamert et al.[22]	22, n/a, n/a	1–14 (3.8) y	1 mo, 6 mo, 12 mo	Auditory performance	No difference

Mito. 12S rRNA, mitochondrial 12S rRNA gene; CI, cochlear implant; IOWA Matrix, IOWA Matrix Level B closed-set sentence test; GASP, Glendonald Auditory Screening Procedure; (IT-)MAIS, (Infant-Toddler) Meaningful Auditory Integration Scale; CAP, Categories of Auditory Performance scale; SIR, Speech Intelligibility Rating scale; n/a, not applicable.

By contrast, certain studies revealed that genetic factors might not be a reliable predictor of CI outcomes [13,19–22]. The discrepancies among these studies may be because different tests were administered to evaluate patients with different lengths of CI use (Table 1). Some studies only addressed the outcomes for the first 2 years after implantation [11,12,15,20,22], while others preformed assessments until postoperative year 3 [13,14,18,19]. None of these studies, however, has reported long-term follow-up results in patients with different types of genetic deafness. To clarify the contribution of genetic factors to the CI outcomes, we investigated the long-term speech and language performance in CI children with mutations in common deafness genes and compared their outcomes to CI children without mutations.

## Materials and Methods

### Participants

A total of 222 patients who received cochlear implantation at a tertiary referral center (Chang-Gung Memorial Hospital) were enrolled in this study. None of them received CIs bilaterally. All subjects were ethnically Han Chinese and had Mandarin Chinese as their native language. After the exclusion of 39 patients who were implanted after the age of 18 years or used the CIs for less than 3 years, speech/language evaluations were administered on 183 patients. All of these patients were implanted with Nucleus CI24R(CS) or Nucleus CI24RE(CA) Freedom, went to mainstream schools and received auditory-verbal rehabilitation after implantation. The study protocol and written informed consent form were approved by the Chang-Gung Memorial Hospital Ethics Committee for Human Studies. All written informed consent forms signed by the participants or the guardians of the underage participants involved in our study were obtained before beginning the testing procedures.

### Genetic examination

All subjects underwent mutation screening for the three common deafness genes, namely *GJB2*, *SLC26A4* and *MTRNR1*, using direct sequencing [18]. Mutation screening included both exons of *GJB2*, all of the 21 exons of *SLC26A4*, and the entire mitochondrial 12S rRNA gene. For patients who carried only one mutant allele in *GJB2* or *SLC26A4*, their DNA samples were further analyzed using two different massively parallel sequencing (MPS) panels to screen for copy number variants or large insertions/deletions in *GJB2* or *SLC26A4*, as well as causative mutations in other deafness genes. Patients with mono-allelic *GJB2* mutations were subjected to an MPS panel which targeted the entire length (i.e. both the coding and non-coding regions) of *GJB2* and the coding regions of 128 known deafness genes, including five other gap junction genes: *GJA1*, *GJB1*, *GJB*3, *GJB4*, and *GJB6* [23–25]. Patients with mono-allelic *SLC26A4* mutations were subjected to another MPS panel targeting 13 genes which had been related to enlarged vestibular aqueduct, and this panel specifically encompassed the entire length of *SLC26A4*, including exons, introns, and untranslated regions. Only patients with confirmative genotypes in the three common deafness genes, i.e. those with two mutant *GJB2* or *SLC26A4* alleles and those with definite *MTRNR1* mutations, were included for further analyses.

### Evaluation of post-implant auditory, speech and language performances

#### Auditory performance and speech intelligibility measures

The Categories of Auditory Performance (CAP) scale and Speech Intelligibility Rating (SIR) scale were rated by speech therapists preoperatively and at post-implant years 3 and 5. The CAP is a nonlinear hierarchical scale that assesses the auditory performance of deaf patients and consists of 8 categories (from 0 to 7; S1 Appendix) [26]. The SIR classifies the intelligibility of patients’ spontaneous speech into 5 categories (from 1 to 5; S1 Appendix) [27]. Higher ratings indicate better performances. The reliability of both scales has been confirmed [26,27].

#### Speech perception measures

Three open-set speech perception tests were administered at post-implant years 3 and 5: an easy sentence test, a difficult sentence test and a phonetically balanced (PB) monosyllabic word test. The two sentence tests were developed according to the Central Institute for the Deaf (CID) Everyday Sentence test [28]. The easy sentence test consists of 15 sentences that include 1–7 key words frequently used in daily conversations, e.g., "book" (S2 Appendix). The difficult sentence test has 20 sentences, each with 1–10 key words with lower familiarity, e.g., "examine" (S3 Appendix). The PB word test contains 25 monosyllabic words (S4 Appendix) [29]. The subjects needed to verbally repeat the word/sentence spoken by the test conductor, who spoke each word/sentence with the mouth covered to prevent lip-reading. The subjects were scored based on the number of (key) words correctly repeated, which was converted to percentages for further analysis.

#### Language skill measures

At post-implant year 5, the Revised Primary School Language Assessment [30] was used to assess receptive and expressive language abilities (see Wu et al. [31] for details). The tests were given orally to the subjects. Raw scores were transformed into T scores based on the age-matched normal-hearing normative sample provided by the test developer (mean = 50 ± 10) [30].

### Statistical analysis

Statistical analyses were conducted using SPSS software (version 17.0; SPSS; SPSS, Inc., Chicago, IL, USA). Descriptive statistical analyses were applied on genotypes and mutant alleles using frequency measurements. The Mann-Whitney *U* test was utilized to make between-group comparisons of test results. The Kruskal-Wallis test was conducted to compare the test results of more than two groups. A *p* value of less than 0.05 was considered significant. For post hoc Mann-Whitney *U* tests, the Bonferroni correction was used to adjust the *p* values of multiple comparisons.

## Results

Of the 183 CI recipients, four had mono-allelic *GJB2* mutations, three had mono-allelic *SLC26A4* mutations, and five had the m.961delT variant. These patients (n = 12) were excluded because of non-confirmative genotypes, leaving 171 cases to be analyzed. Forty-eight (26.2%) were found to have confirmative mutations in the common deafness genes (hereafter called "the mutation group," see Tables 2 and 3), including 25 cases with 2 mutated alleles in *GJB2* (52.1% of the 48 implantees), 23 with 2 mutated alleles in *SLC26A4* (47.9%), and 0 with mutations in the mitochondrial 12S rRNA gene (see Table 4). No mutations were detected in the common deafness genes in the remaining 123 patients ("the no-mutation group").

**Table 2:** Genotypes of the 48 children with mutations in common deafness genes.

Genotype	Numbers of patients
*GJB2*	
c.235delC/c.235delC	12
p.V37I/p.V37I	5
c.235delC/c.299_300del	2
p.V37I/c.235delC	1
p.V37I/p.R143W	1
p.V37I/p.R143Q^a^	1
c.176_191del/c.235delC	1
p.W77X/c.235delC	1
c.235delC/c.511_512del	1
Total	25
*SLC26A4*	
c.919-2A>G/c.919-2A>G	12
c.919-2A>G/p.A372V	3
c.919-2A>G/p.H723R	2
p.P8T/p.P8T^b^	1
c.416-1G>A/c.919-2A>G	1
c.916dup/c.919-2A>G	1
c.919-2A>G/c.974_977delinsTTAAATTA	1
c.919-2A>G/p.Q696X	1
p.K369X/p.T410M	1
Total	23^c^

^a^ The phenotype of SNHI might be caused either by bi-allelic *GJB2* mutations or by the *GJB2* p.R143Q mutation alone in dominant inheritance.

^b^ The p.P8T (c.22C>A) mutation is in a minor *SLC26A4* transcript (UCSC Genes:uc011kmb.2).

^c^ Six of the 23 patients with bi-allelic *SLC26A4* mutations also incidentally carry one *GJB2* variant allele, including 3 with *GJB2* p.V37I, 1 with *GJB2* c.235delC, 1 with *GJB2* c.299_300del, and 1 with *GJB2* c.508_511dup.

**Table 3:** Demographic information of 171 subjects with and without mutations in *GJB2*, *SLC26A4* and the mitochondrial 12S rRNA gene.

	With mutations	No mutations	All subjects
Number of subjects	48	123	171
Gender (male/female)	24 (50%) / 24 (50%)	61 (50%) / 62 (50%)	85 (50%) / 86 (50%)
Pre-op use of HA (yes/no)	46 (96%) / 2 (4%)	118 (96%) / 5 (4%)	164 (96%) / 7 (4%)
Unilateral/bilateral CI	48 (100%) / 0 (0%)	122 (99%) / 1 (1%)	170 (99%) / 1 (1%)
Chronological age (years)	12.0 ± 4.1	11.6 ± 4.5	11.7 ± 4.4
Age at detection of HL (years)	1.7 ± 1.0	1.6 ± 1.3	1.7 ± 1.3
Age at implantation (years)	4.4 ± 2.4	4.1 ± 2.8	4.2 ± 2.7
Duration of implant use (years)	7.6 ± 3.5	7.5 ± 3.4	7.5 ± 3.4

Pre-op, preoperative; HA, hearing aid; CI, cochlear implant; HL, hearing loss.

**Table 4:** Number of subjects with mutations in *GJB2*, *SLC26A4* and the mitochondrial 12S rRNA gene.

			All subjects		
Genotype	CI before 3.5 y	CI after 3.5 y	N	AgeHL	AgeCI
*GJB2*	16	9	25	1.5 ± 0.8	3.5 ± 1.8
*SLC26A4*	6	17	23	2.0 ± 1.2	5.4 ± 2.6
Mito. 12S rRNA	0	0	0	n/a	n/a
No mutations detected	75	48	123	1.6 ± 1.3	4.1 ± 2.8
All subjects	97	74	171	1.7 ± 1.3	4.2 ± 2.7

CI, cochlear implantation; AgeHL, age at detection of hearing loss; AgeCI, age at implantation; Mito. 12S rRNA, mitochondrial 12S rRNA gene; n/a, not available.

The age of 3.5 years was used as the cutoff point for early-late implantation, which was determined based on the literature [32,33]. Of note, as many as 74 (43%) of the 171 cases were classified as late-implantation. The average ages at detection of HL and implantation in the 171 patients were 1.7 y and 4.2 y, respectively (Table 3). A possible explanation for the late detection and implantation is that the coverage rate of newborn hearing screening in Taiwan had not increased to 90% until 2012 [34], resulting in delayed diagnosis in certain hearing-impaired children.

A total of 109 patients (31 with mutations, 78 without mutations) had used the CIs for more than 5 years and thus received evaluations at post-implant year 5.

### Comparison between CI recipients with and without genetic mutations

No significant difference was found between the mutation group and the no-mutation group regarding their age at implantation (4.4 ± 2.4 years vs. 4.1 ± 2.8 years, p > 0.05) and duration of implant use (7.6 ± 3.4 years vs. 7.5 ± 3.4 years, p > 0.05). Their CAP scores at the pre-implant visit (U = 1691.5, p < 0.001), post-implant year 3 (U = 2062.5, p = 0.002) and post-implant year 5 (U = 623.0, p = 0.027) were significantly different, and so were their SIR scores at the three test time points (respectively: U = 2225.0, p = 0.013; U = 2099.0, p = 0.004; U = 620.0, p = 0.022; see Table 5).

**Table 5:** CAP and SIR scores in all subjects, subjects implanted before the age of 3.5 years and subjects implanted after 3.5 years.

	CAP			SIR		
Subjects	Pre-op	Post-op 3 y	Post-op 5 y	Pre-op	Post-op 3 y	Post-op 5 y
All subjects (n = 171)						
With mutations (n = 48)	2.0	6.0	6.0	1.0	4.0	5.0
No mutations (n = 123)	0.5	6.0	6.0	1.0	4.0	5.0
*P* value*	< 0.001	0.002	0.027	0.013	0.004	0.022
CI before 3.5 y (n = 97)						
With mutations (n = 22)	1.0	6.0	6.5	1.0	4.0	5.0
No mutations (n = 75)	0.0	5.0	6.0	1.0	3.0	4.0
*P* value*	0.017	0.001	0.035	0.046	0.004	0.038
CI after 3.5 y (n = 74)						
With mutations (n = 26)	2.5	6.0	6.0	2.0	4.0	5.0
No mutations (n = 48)	1.0	6.0	6.0	2.0	4.0	5.0
*P* value*	0.012	> 0.05	> 0.05	> 0.05	> 0.05	> 0.05

CI, cochlear implant; Pre-op, preoperative; Post-op, postoperative; CAP, Categories of Auditory Performance scale; SIR, Speech Intelligibility Rating scale.

* Compared with "No mutations" using Mann-Whitney *U* test.

Among patients who received implantation before age 3.5 years, the mutation group had significantly higher CAP and SIR scores than the no-mutation group at the pre-implant visit (U = 564.5, p = 0.017 for CAP; U = 636.0, p = 0.046 for SIR), post-implant year 3 (U = 471.0, p = 0.001 for CAP; U = 499.5, p = 0.004 for SIR) and post-implant year 5 (U = 159.5, p = 0.035 for CAP; U = 160.5, p = 0.038 for SIR; Table 5). On the contrary, among patients implanted after the age of 3.5 years, significant differences between the mutation and no-mutation groups were noted only in the pre-implant CAP scores (U = 388.5, p = 0.012; Table 5).

On the three speech perception tests (i.e., easy sentence, difficult sentence and PB word) and the receptive and expressive language tests, patients with and those without mutations obtained similar scores without significant differences at the two post-implant test time points, regardless of their age at implantation (all p > 0.05; Table 6).

**Table 6:** Speech perception and language skill measures in all subjects, subjects implanted before the age of 3.5 years and subjects implanted after 3.5.

	Post-op 3 y			Post-op 5 y				
Subjects	Easy sentence	Difficult sentence	PB word	Easy sentence	Difficult sentence	PB word	RL	EL
All subjects (n = 171)								
With mutations (n = 48)	80.1 ± 26.2	77.5 ± 24.3	79.9 ± 19.8	90.4 ± 21.6	83.6 ± 24.5	79.3 ± 24.0	42.1 ± 16.1	49.2 ± 13.7
No mutations (n = 123)	75.7 ± 29.2	69.5 ± 30.5	72.7 ± 26.2	82.3 ± 25.0	77.7 ± 25.1	72 ± 27.6	43.4 ± 16.2	50.7 ± 12.9
*P* value*	0.613	0.328	0.336	0.133	0.224	0.260	0.808	0.777
CI before 3.5 y (n = 97)								
With mutations (n = 22)	84.6 ± 20.7	77.9 ± 20.4	86.7 ± 9.4	89.8 ± 29.5	85.1 ± 30.1	87.6 ± 15.7	47.1 ± 12.7	54.8 ± 8.7
No mutations (n = 75)	72.1 ± 29.1	62.8 ± 31.2	75.5 ± 22.0	90.7 ± 14.3	83.5 ± 19.1	84.4 ± 12.0	45.6 ± 13.3	51.5 ± 11.7
*P* value*	0.128	0.143	0.132	0.226	0.228	0.256	0.488	0.468
CI after 3.5 y (n = 74)								
With mutations (n = 26)	76.7 ± 29.8	77.1 ± 27.9	74.8 ± 24.0	91 ± 10.6	81.9 ± 18.0	69.0 ± 29.3	36.4 ± 18.2	42.9 ± 15.9
No mutations (n = 48)	80.3 ± 29.2	79.6 ± 27.1	68.2 ± 32.1	69.6 ± 31.9	68.9 ± 30.7	53.7 ± 33.7	38.9 ± 20.7	48.9 ± 15.4
*P* value*	0.321	0.673	0.854	0.097	0.358	0.325	0.533	0.371

CI, cochlear implant; Post-op, postoperative; Easy sentence, easy-sentence perception test; Difficult sentence, difficult-sentence perception test; PB word, phonetically balanced monosyllabic word perception test; RL, receptive language skill test; EL, expressive language skill test.

* Compared with "No mutations" using Mann-Whitney *U* test.

### Comparison between different genotypes

The performances in children specifically with *GJB2* (n = 25) or *SLC26A4* mutations (n = 23) were further compared to those without mutations. There was no significant difference in age at detection of hearing loss between the three groups (p > 0.05; Table 4), but their ages at implantation differed significantly (p = 0.010), with the *SLC26A4* group being significantly later implanted than the other two groups (p = 0.003 and p = 0.005 for post-hoc comparisons with the *GJB2* group and the no-mutation group, respectively, where the significance was reached when p < α/3 = 0.017 for multiple comparisons). The later implantation in the *SLC26A4* group probably could be explained by the progressive or fluctuating hearing loss associated with *SLC26A4* mutations. In contrast to patients with *GJB2* mutations who usually demonstrate congenital severe to profound SNHL, patients with *SLC26A4* mutations often start with milder SNHL at the time of diagnosis which does not progress to profound SNHL necessitating cochlear implantation until a later age.

For children implanted before the age of 3.5 years, there was a significant difference between children with *GJB2* or *SLC26A4* mutations and those without mutations in CAP scores at the pre-implant visit (p = 0.038), post-implant year 3 (p = 0.005) and post-implant year 5 (p = 0.012), as well as in SIR scores (p = 0.012) and easy sentence scores (p = 0.048) at post-implant year 3. Post hoc tests showed that patients with *GJB2* mutations had significantly higher CAP scores than those without mutations at post-implant year 3 (p = 0.010; Table 7). Patients with *SLC26A4* mutations performed significantly better than the no-mutation group did on the easy sentence perception test at post-implant year 3 (p = 0.016) and on the CAP scale at post-implant year 5 (p = 0.004). No significant differences were found between patients with *GJB2* mutations and those with *SLC26A4* mutations, regardless of the test type or the test time point (all p > 0.017). Except for the easy-sentence test, none of the other measures of speech perception and language skills showed significant differences between the three groups (all p > 0.017).

**Table 7:** CAP and SIR scores in subjects broken down by age at implantation (before and after 3.5 years) and genotypes (*GJB2*, *SLC26A4* and no mutations).

		Pre-op		Post-op 3 y			Post-op 5 y	
Age at CI	Genotype	CAP	SIR	CAP	SIR	Easy sentence	CAP	SIR
CI before 3.5 y	*1*. *GJB2* (n = 16)	1	1	6	4	79.4 ± 22.3	6	5
	*2*. *SLC26A4* (n = 6)	2	1	6	4.5	98.0 ± 2.8	7	5
	*3*. No mutations (n = 75)	0	1	5	3	72.1 ± 29.1	6	4
	*P* value (1 vs. 2)*	> 0.017	> 0.017	> 0.017	> 0.017	> 0.017	> 0.017	> 0.017
	*P* value (1 vs. 3)*	> 0.017	> 0.017	0.01	> 0.017	> 0.017	> 0.017	> 0.017
	*P* value (2 vs. 3)*	> 0.017	> 0.017	> 0.017	> 0.017	0.016	0.004	> 0.017
CI after 3.5 y	*1*. *GJB2* (n = 9)	1	1	6	4	62.0 ± 26.5	6	4.5
	*2*. *SLC26A4* (n = 17)	4	3	6	5	83.1 ± 29.6	7	5
	*3*. No mutations (n = 48)	1	2	6	4	80.3 ± 29.2	6	5
	*P* value (1 vs. 2)*	0.015	0.007	> 0.017	> 0.017	> 0.017	> 0.017	> 0.017
	*P* value (1 vs. 3)*	> 0.017	> 0.017	> 0.017	> 0.017	> 0.017	> 0.017	> 0.017
	*P* value (2 vs. 3)*	0.004	> 0.017	> 0.017	> 0.017	> 0.017	> 0.017	> 0.017

CI, cochlear implant; Pre-op/post-op, preoperative/postoperative; CAP, Categories of Auditory Performance scale; SIR, Speech Intelligibility Rating scale; Easy sentence, easy-sentence perception test.

* Compared using the Mann-Whitney *U* test as a post hoc test; significance was reached when p < α/3 = 0.017 (Bonferroni correction) for multiple comparisons of the three groups (*GJB2*, *SLC26A4* and no mutations).

Regarding children who received CIs after 3.5 years, significant differences were noted only in pre-implant CAP and SIR scores (p = 0.009 and p = 0.039, respectively) between children with *GJB2* mutations, children with *SLC26A4* mutations and those without mutations. No significant differences could be found at post-implant years 3 and 5 between the three groups. As post hoc tests showed (see Table 7), patients with *SLC26A4* mutations obtained significantly better pre-implant CAP scores than the *GJB2* group (p = 0.015) and the no-mutation group (p = 0.004). The *SLC26A4* group also demonstrated significantly better pre-implant SIR scores than the *GJB2* one (p = 0.007). The three groups did not perform differently on any of the speech perception and language skill tests (all p > 0.017).

## Discussion

Our results revealed that for children who received CIs before the age of 3.5 years, patients with mutations in common deafness genes, including *GJB2* and *SLC26A4*, demonstrated significantly better long-term auditory performance and speech intelligibility at post-implant year 3 and 5 than those without mutations. By contrast, for children who received CIs after 3.5 years, no differences were observed in post-implant outcomes between patients with and without mutations.

Early-implanted patients with mutations demonstrated better post-implant outcomes than those without mutations, most likely because the pathogenic consequences of *GJB2* and *SLC26A4* mutations are confined to the cochlea, sparing the neural integrity of the auditory system that is crucial for the function of CIs [17,35]. Despite the fact that both groups were implanted before 3.5 years of age, patients with *GJB2* or *SLC26A4* mutations, benefitting from the intact auditory pathway, have a better chance of developing satisfactory outcomes after implantation than those with unknown etiologies, where the causes of deafness are greatly heterogeneous.

This neural integrity, however, did not seem to play a significant role in the postoperative outcomes when patients were implanted after the age of 3.5 years. No significant differences could be noted in the post-implant outcomes between later-implanted patients with and those without mutations. The only difference between the two groups lies in their pre-implant auditory performance (median CAP score 2.5 vs. 1, p = 0.012; see Table 5), which is most likely because more than half (n = 17) of the later-implanted patients in the mutation group had *SLC26A4* mutations. Recessive mutations in *SLC26A4* are responsible for non-syndromic enlarged vestibular aqueduct (EVA) [36] and Pendred syndrome [37], which are often associated clinically with progressive or fluctuating hearing loss [38]. As a consequence, patients with *SLC26A4* mutations might acquire more hearing experiences before implantation than those with congenital hearing loss. The larger amount of pre-implant hearing experiences thus led to higher pre-implant CAP scores in the *SLC26A4* group than in *GJB2* group and no-mutation group, which in turn resulted in higher pre-implant CAP scores in the mutation group than in the no-mutation group. However, the effect of previous hearing experiences became less visible with an increased length of implant use. After 3 years of use, all patients implanted after the age of 3.5 years produced similar outcomes.

As far as we know, only three studies have correlated the CI outcomes to the *SLC26A4* genotypes in the literature [11,17,18]. Consistent with the current findings, our previous studies [17,18] showed better results in patients with *SLC26A4* mutations than those without genetic etiologies, while Yan et al. [11] did not find significant differences between the two groups. The discrepancy between studies probably results from different durations of follow-up periods, as Yan et al. [11] focused on short-term outcomes at post-implant years 1 and 2.

A previous study of ours showed that SIR scores in patients implanted before the age of 5 years did not plateau until 5 years of implant use [39], indicating that children who receive CI early need 5 years to develop mature speech skills after implantation. By documenting the long-term outcomes in a large CI cohort, the present study further revealed that neither patients with *GJB2* mutations nor patients with *SLC26A4* mutations performed differently from those without mutations on the SIR scale after 5 years of use (Table 7). Their performances on speech perception and language skills tests were also similar to those without mutations. This is in line with our previous study on EVA that patients with and without EVA obtained comparable outcomes after using CIs for more than 5 years [40]. Taken together, although the outcomes with CIs could be influenced by the etiologies of deafness, it is likely that the effects of the etiologies are eventually diluted after 5 years of implant use. High levels of performance (i.e., median CAP = 6, median SIR = 5 and mean speech perception scores > 70%) were reached at post-implant year 5, regardless of the etiologies.

The decreased effect of genetic mutations on CI outcomes after several years of implant use may again be associated with neural integrity. On the one hand, common deafness-related genes such as *GJB2* and *SLC26A4* have minimal influence on the integrity of spiral ganglion neurons, leading to the speedy restoration of hearing after cochlear implantation. On the other hand, cases with unknown etiologies are more likely to have degenerated spiral ganglion neurons, which could result in slower post-implant development. However, considering that children with and without mutations both received aural-verbal rehabilitation after implantation, it is still possible for the no-mutation group to develop their skills steadily and catch up with the mutation group after using CIs for some years. The gap between the mutation group and the no-mutation group thus becomes smaller with the increased length of use.

It seems that the correlation between genetic diagnosis and CI outcomes is greatly influenced by other factors, including age at implantation, duration of implant use, and types of outcome measurements. This may account for the conflicting results among previous studies in the literature (Table 1), as their observations were made on the basis of different study designs and settings. Therefore, these factors should be taken into consideration to avoid potential biases in the interpretation when the relationship between genetic diagnosis and CI outcomes is explored. In the current study, it appears that the contribution of common genetic mutations is modulated by age at implantation and duration of CI use. Genetic mutations have an impact on post-implant outcomes only when the patients received CIs early (i.e., before the age of 3.5 years), and the impact becomes weaker after 5 years of implant use.

Recently, massively parallel sequencing (MPS) has been proven to be a powerful tool for genetic examination in hearing loss [41]. Theoretically, mutations in different deafness genes lead to different pathologies and might result in varied CI outcomes. Using the MPS technology, it has been demonstrated that mutations in the *TMPRSS3* gene were associated with poor CI outcome [35], whereas mutations in the *MYO15A*, *TECTA*, and *ACTG1* genes also showed relatively good auditory performance after implantation [42]. Our recent study [23] added that mutations in the *PCDH15* and *DFNB59* genes were associated with poor CI performance, but children with these mutations might demonstrate clinical features indistinguishable from those of other typical pediatric CI candidates before operation. Accordingly, the inclusion of a comprehensive genetic examination into the pre-CI evaluation battery could be anticipated in the near future, as it provides critical information for determining appropriate rehabilitation programs and setting the expectations of physicians, audiologists, schools, and families.

## Conclusion

*GJB2* and *SLC26A4* mutations were associated with good post-implant outcomes. However, their effect on CI outcomes was modulated by the age at implantation and the duration of implant use. Patients with *GJB2* or *SLC26A4* mutations showed better post-implant auditory performance and speech intelligibility than those without mutations only when implanted before age 3.5 years. The effect of genetic mutations became weaker with the increase in the length of implant use and was not observed in those who received CIs after the age of 3.5 years. These results provide insight into the contribution of genetic factors to the outcomes of CIs, and might be useful in steering preoperative counseling and appropriate assessments in CI candidates.

## Supporting Information

S1 Appendix Criteria of Categorical Auditory Performance (CAP) and Speech Intelligibility Rating (SIR) scales. (DOC)

S2 Appendix Easy sentence list for the speech perception test (English translation). (DOC)

S3 Appendix Difficult sentence list for the speech perception test (English translation). (DOC)

S4 Appendix Phonetically-balanced monosyllabic word list for the speech perception test. (DOC)
