# Supplementary material for: Long-Term Cochlear Implant Outcomes in Children with GJB2 and SLC26A4 Mutations
Source: PLoS One. 2015 Sep 23;10(9):e0138575. doi: 10.1371/journal.pone.0138575 (PMC4580418; doi:10.1371/journal.pone.0138575)
Supplement: S1 Appendix — (DOC) [file pone.0138575.s001.doc]

**S1 Appendix**. Criteria of Categorical Auditory Performance (CAP) and Speech Intelligibility Rating (SIR) scales.

| Rating Scale | CAP Criteria | SIR Criteria |
| --- | --- | --- |
| 7 | Use of telephone with known listener | n/a |
| 6 | Understanding of conversation without lip-reading | n/a |
| 5 | Understanding of common phrases without lip-reading | Connected speech is intelligible to all listeners. Child is understood easily in everyday contexts. |
| 4 | Discrimination of some speech sounds without lip-reading | Connected speech is intelligible to a listener who has a little experience of a deaf person’s speech |
| 3 | Identification of environmental sounds | Connected speech is intelligible to a listener who concentrates and lip-reads |
| 2 | Response to speech sounds | Connected speech is unintelligible. Intelligible speech is developing in single words when context and lip-reading cues are available |
| 1 | Awareness of environmental sounds | Connected speech is unintelligible. Pre-recognizable words in spoken language, primary mode of communication may be manual |
| 0 | No awareness of environmental sounds | n/a |

n/a, not applicable.
