# Supplementary material for: Long-Term Cochlear Implant Outcomes in Children with GJB2 and SLC26A4 Mutations
Source: PLoS One. 2015 Sep 23;10(9):e0138575. doi: 10.1371/journal.pone.0138575 (PMC4580418; doi:10.1371/journal.pone.0138575)
Supplement: S2 Appendix — (DOC) [file pone.0138575.s002.doc]

**S2 Appendix. Easy sentence list for the speech perception test (English translation).** The key words are underlined.
(1) This book is good.
(2) Have you brought all your stuff?
(3) Be careful when driving the car.
(4) It does not matter.
(5) Turn off the television.
(6) Where is the newspaper?
(7) Excuse me, is the boss there?
(8) That coat looks really good.
(9) I am having a headache.
(10) Tomorrow is a holiday. Do you want to go out?
(11) It seems that I am having a fever.
(12) Please don’t smoke.
(13) The weather is quite nice today.
(14) How many brothers and sisters do you have?
(15) How many people are there in your family?
