# Supplementary material for: Long-Term Cochlear Implant Outcomes in Children with GJB2 and SLC26A4 Mutations
Source: PLoS One. 2015 Sep 23;10(9):e0138575. doi: 10.1371/journal.pone.0138575 (PMC4580418; doi:10.1371/journal.pone.0138575)
Supplement: S3 Appendix — (DOC) [file pone.0138575.s003.doc]

**S3 Appendix. Difficult sentence list for the speech perception test (English translation).** The key words are underlined.
(1) Can you hear what he is saying?
(2) How is your health condition?
(3) How about it?
(4) We will go there right after having dinner.
(5) Who is in there?
(6) Very busy.
(7) This movie is very good.
(8) We often go to the park.
(9) This building is taller than that one.
(10) The doctor comes to carry out examinations on him every day.
(11) Many books are put on the table.
(12) I am so tired.
(13) Is sister busily working?
(14) I am a student.
(15) That novel is very interesting.
(16) There will be a movie today evening.
(17) He has graduated for about two years.
(18) When he is anxious, he cannot even speak a word.
(19) Sit down, please!
(20) It is summer now.
