# Supplementary material for: Long-Term Cochlear Implant Outcomes in Children with GJB2 and SLC26A4 Mutations
Source: PLoS One. 2015 Sep 23;10(9):e0138575. doi: 10.1371/journal.pone.0138575 (PMC4580418; doi:10.1371/journal.pone.0138575)
Supplement: S4 Appendix — (DOC) [file pone.0138575.s004.doc]

**S4 Appendix. Phonetically-balanced monosyllabic word list for the speech perception test.** Pinyin characters are used. English translations are parenthesized.

| bà (dad) | zhǐ (only) | nǔ (effort) | ling (bell) | qì (air) |
| --- | --- | --- | --- | --- |
| nioú (cow) | zuǒ (left) | jìn (nearby) | yén (salt) | gòu (enough) |
| jiěn (minus) | fù (father) | kāi (open) | zhú (bamboo) | rù (enter) |
| jiǎng (talk) | cīng (star) | lí (pear) | zhè (this) | zhàn (battle) |
| yā (duck) | yào (want) | yú (fish) | jiěh (sister) | guó (nation) |
